# Efficacy of the New Double-Layer Stent for Unresectable Distal Malignant Biliary Obstruction: A Single-Center Retrospective Study

**DOI:** 10.1155/2012/680963

**Published:** 2012-03-07

**Authors:** Ken Ito, Yoshinori Igarashi, Takahiko Mimura, Yui Kishimoto, Yoshinori Kikuchi, Naoki Okano

**Affiliations:** Division of Gastroenterology and Hepatology, Toho University Omori Medical Center, 6-11-1, Omori Nishi, Ota-ku, Tokyo 143-8541, Japan

## Abstract

*Background and Aims*. For distal malignant biliary obstruction in cases with short life expectancy, occlusion of plastic stents (PSs) does not usually occur before death, and the application of such a procedure is considered adequate from the viewpoint of cost-effectiveness. *Methods and Setting*. A new commercially available DLS with side holes, a conventional DLS, and, uncovered self-expanding metal stents (SEMSs) were retrospectively evaluated in patients with jaundice due to unresectable distal malignant biliary obstruction. *Results*. A total of 64 patients received endoscopic biliary stenting (23 patients with the new DLS, 24 patients with conventional DLS, and 17 patients with uncovered SEMS) from December 2002 to August 2009. Median patency time was found to be 198 days for the new DLS group and 99 days for the conventional DLS group, revealing a significant difference between devices. There was, however, no significant difference in median patency time between the new DLS and the uncovered SEMS (198 days versus 344 days). *Conclusion*. The new DLS is efficient and safe and may be considered the first choice for unresectable distal malignant obstruction in cases with short life expectancy.

## 1. Introduction

Endoscopic biliary stenting is a well-established method, and endoscopic palliation of malignant jaundice can improve patient quality of life. Two types of biliary stents, each of a different material, are routinely used: plastic stents (PSs) and self-expanding metal stents (SEMSs), and it is necessary for each stent to be appropriately selected for each case.

SEMSs have a longer patency period than plastic stents. However, uncovered SEMSs (UMSs) also have several drawbacks, including higher cost, irretrievability once placed, and susceptibility to tumor overgrowth and ingrowth, which may result in stent dysfunction [1].

**Figure 1:**
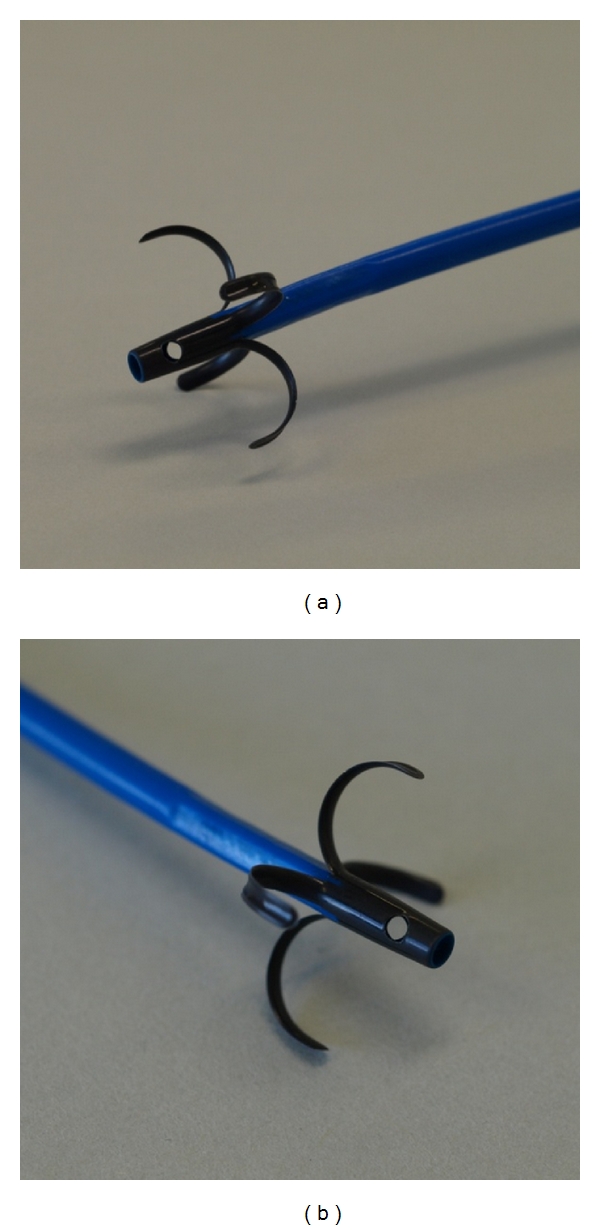
DLS with side holes (Olympus Medical Systems, Tokyo, Japan). Two side holes (18-gauge size) on the proximal side of the tip.

PSs on the other hand are less expensive and remain a popular alternative to metallic stents. Their major drawback, however, is their propensity to clog, often necessitating repeated stent changes in order to maintain biliary drainage [2]. To prolong the duration of PSs patency, a double-layer stent (DLS; Olympus Medical Systems, Tokyo, Japan) has been developed and shown to be superior to regular PSs with respect to patency and is consequently being used more frequently than PSs [3]. When bending of the malignant bile duct is advanced, the tip of the stent impacts the biliary wall, and since the stent has an orifice only in the tip, this situation leads to an early occlusion of DLS and becomes a major issue [3, 4]. To handle this issue appropriately, we modified the DLS with two holes (18 gauge) on the proximal side of the tip (Figure 1) and found that bile duct impaction was reduced and stent patency was low. The new DLS was authorized for use under the Pharmaceutical Affairs Act in April 2010. Therefore, we retrospectively analyzed the efficacy and the safety of this new, commercially available stent.

## 2. Subjects

A total of 64 obstructive jaundice patients with unresectable malignant lower biliary obstruction received endoscopic biliary stenting (EBS) at the Toho University Omori Medical Center (Table 1). Endoscopic retrograde cholangiopancreatography (ERCP) was performed using a large channel (4.2 mm, 3.7 mm) duodenoscope (Olympus TJF-260V/TJF-240, JF-260V; Olympus Medical Systems). Endoscopic sphincterotomy (EST) was routinely performed before stent insertion. After EST, a 7.2Fr endoscopic nasobiliary drainage (Hanaco Medical Co., Saitama, Japan) or 7Fr PSs (Zimmon type biliary stent; Wilson-Cook Medical, Winston-Salem, NC) was temporarily inserted. After confirming an improvement of serum total bilirubin (<3 mg/dL) level, a conventional DLS, new DLS, or UMS was inserted.

With regard to the UMS group, because we experienced a case in 2001 in which covered SEMSs (CMS) exhibited migration, UMS has since been used for malignant lower biliary obstruction. For UMS, a Zilver stent (Wilson-Cook Medical) was inserted because of its very low axial force values [5].

We gave priority to placing DLS for distal malignant biliary obstruction cases with short life expectancy from December 2002. Because a severely bending bile duct impacted on the proximal stent orifice in a manner similar to that of bile duct invasion, the stent had to be replaced after a short time due to its short patency period. There were however cases of severely bending bile duct due to bile duct invasion, for which we had to replace DLS because the proximal side of DLS was in contact with the bending bile duct. Accordingly, we gave priority to placing uncovered SEMSs from February 2004. To improve the plastic stent, we selected the new modified DLS with two holes on the proximal side and gave priority to placing the new DLS from January 2005 to September 2009.

Chemotherapy was performed in all cases, except in those that were expected to have poor prognosis within three months, elderly cases, and cases of low performance status. For cases of unresectable pancreatic cancer, gemcitabine (GEM) was administrated in a standard manner (drip infusion of 1000 mg/m^2^ at a stable speed) once per week for three consecutive weeks, with a one-week interval between courses. After confirming a decrease of 3.0 mg/dL in serum total bilirubin level, GEM was started. If adverse events occurred, administration of GEM was postponed or its dose was reduced [6, 7]. Chemotherapy for the metastasis group was based on the primary disease. Adverse effects were evaluated following the National Cancer Institute Common Terminology Criteria for Adverse Events ver. 3.0 (CTCAE) [8].

## 3. Examination Items

Obstruction periods, causes of obstruction, complications related to stent insertion, and the status of GEM administration in cases of malignant lower biliary obstruction were examined. Informed consent was obtained from all patients. This study was conducted following approval by the institutional review board of our hospital.

## 4. Exclusion Items

A history of biliary surgery, accidental symptoms related to ERCP, and endoscopic percutaneous or surgical drainage, as well as papilla cancer, were determined as exclusion items. Moreover, the patients in the conventional DLS group that was investigated in the Japanese Multicenter Randomized Trial of Endoscopic Stenting for nonresection pancreatic head cancer (JM-TEST [9]) from October 2005 to October 2007 were also excluded.

## 5. Statistical Analysis

Statistical analysis was performed using SPSS for Windows, version 11.0J (SPSS Inc., Chicago, IL). All continuous variables are presented as mean ± standard error. A *P* value of <0.05 was considered statistically significant. Comparisons of the outcome variable (stent clogging) were analyzed using the chi-square test or Fisher exact test. The cumulative clogging-free survival period was compared across the study groups using the Kaplan-Meier method, and differences in the patency rates were analyzed using the log-rank test. In addition, the effect of stent type on clogging-free survival was analyzed by constructing age-adjusted (≥65 years old) and sex-adjusted Cox proportional hazard regression models with simultaneous introduction of covariates. For all analyses, patient data were censored at the time of stent clogging or death, as well as at stent migration.

The primary endpoint was the interval between stent insertion and the first episode of clogging or migration, or the presence of jaundice at death without stent exchange.

## 6. Results

Followup data until death or stent occlusion were obtained from all new DLS and conventional DLS UMS participants. The period from insertion to obstruction was considered uncensored while the period from insertion until death without obstruction was considered censored.

In the case of distal malignant biliary obstruction, median patency time was 198 days for the new DLS group, 99 days for the conventional DLS group, and 344 days for the UMS group, and a significant difference was noted (log-rank test; *P* = 0.0014; Figure 4). In the GEM group, the median patency time was 245 days for the new DLS group and 95 days for the conventional DLS group, 344 days for the UMS group, and significant difference was noted (log-rank test; *P* = 0.0058; Figure 5). In the non-GEM group, the median patency time was 127 days for the new DLS group, 100 days for the conventional DLS group, and 139 days for the UMS group, and no significant difference was noted (log-rank test; *P* = 0.179; Figure 5).

**Figure 2:**
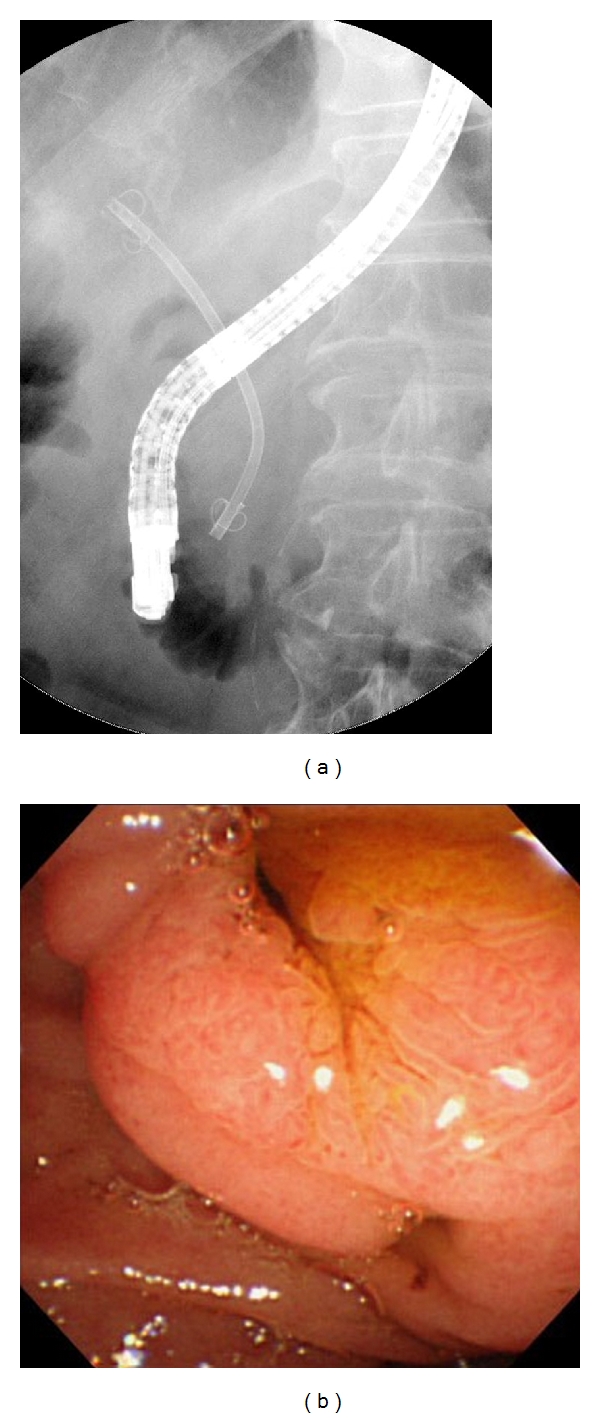
ERCP shows malfunctioning DLS migration at 198 days after placement.

**Figure 3:**
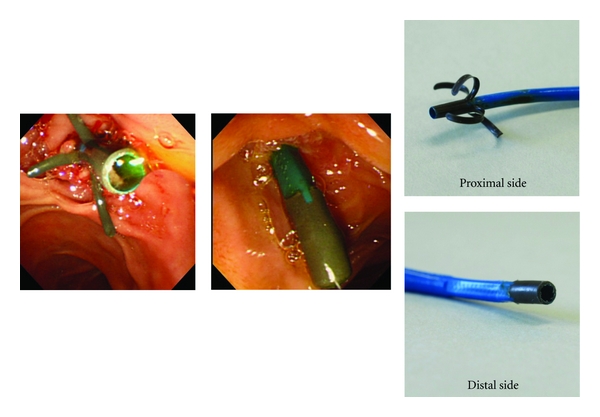
New DLS flap damage at 97 days after placement.

**Figure 4:**
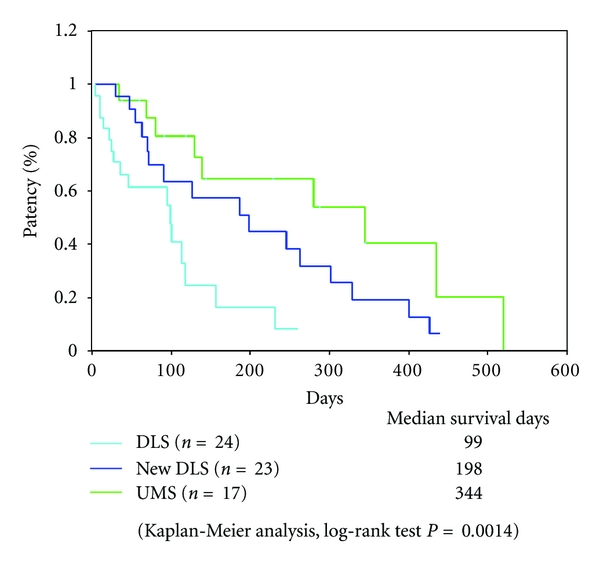
Patency period in malignant lower biliary obstruction with new DLS, conventional DLS, and UMS. Significant difference was noted using the new DLS (log-rank test; *P* = 0.0014).

**Figure 5:**
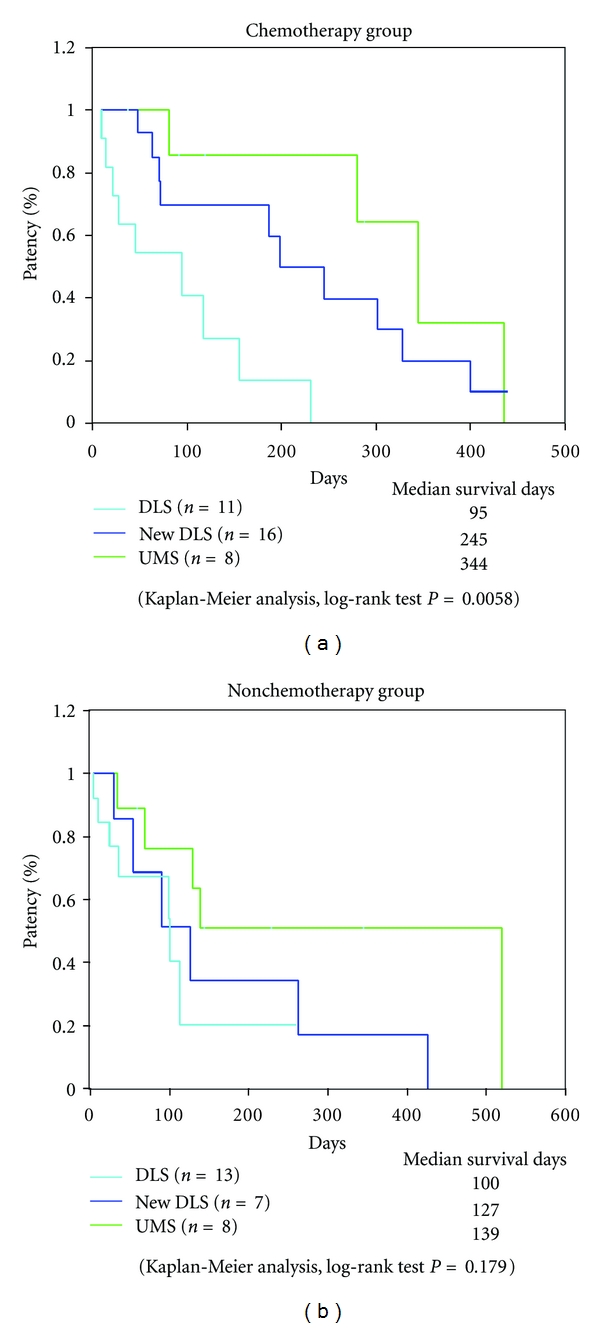
Stent patency in malignant lower biliary obstruction with chemotherapy. Chemotherapy group (a); 245 days for the new DLS group, and 95 days for the conventional DLS group, 344 days for the UMS group (log-rank test; *P* = 0.0058). Nonchemotherapy group (b); 127 days for the new DLS group, 100 days for the conventional DLS group, and 139 days for the UMS group (log-rank test; *P* = 0.179).

Table 2 shows the incidence of stent malfunction. Mean followup was  160 ± 136  days for the new DLS group and  74 ± 66  days for the conventional DLS group (*P* = 0.015). No difference was observed in terms of early stent malfunction (15 side-hole DLSs versus 16 conventional DLSs; *P* = 0.580). Bile duct impaction was found in 3 side-hole DLS patients after 71, 90, and 187 days, and in 3 conventional DLS patients after 10, 14, and 22 days. Cholecystitis was found in 2 conventional DLS patients after 3 and 38 days. Stent migration was found in 1 new DLS patient with metastatic lymphadenopathy, and cholangitis occurred 198 days after placement. ERCP revealed stent migration, and the stent was removed by balloon catheter (Multi3, Olympus Medical Systems) deflation (Figure 2). In one patient, side-hole DLS flap damage on the duodenal side was seen 97 days after placement (Figure 3). Results are summarized in Table 2.

Regarding incidence of stent malfunction, the difference between the new DLS group and the UMS group was not significant (Table 2). Furthermore, univariate analysis revealed no significant differences for nonobstruction factors (Table 3).

## 7. Discussion

Patients with inoperable malignant obstruction of the distal bile duct can be palliated by endoscopic biliary stenting in up to 95% of cases, with a mortality rate of 2% [10, 11]. The mean life expectancy of these patients is short, and to improve quality of life, a palliative stent should ideally remain patent until death. Although polyethylene, Teflon, and polyurethane are often used for PSs, no differences in patency have been found [12, 13]. During the past decade, many randomized clinical trials have been conducted with the goal of optimization stent design. Although smoothness of the stent wall has been considered an important factor for stent function, no detailed quality analysis of the commercially available stents used in the various trials has been performed [13–15]. It has been reported, however, that the presence of side holes increases the amount of sludge in PSs, and therefore, improved TANNENBAUM type stents do not have such side holes. 

Coene et al. showed that prostheses without side holes exhibited significantly lower sludge deposition when compared with those with side holes [13]. Consequently, Seitz et al. demonstrated the efficacy of the 10Fr. Teflon straight stent (e.g., TANNENBAUM stent) without side holes by showing its significantly extended patency [13, 16]. Additional studies have found no significant advantage in stent patency between TANNENBAUM and standard polyethylene stents [17, 18]. To overcome the stent patency problem, the DLS was developed and comprised an inner layer of perfluoroalkoxy (PFA) containing specially processed and chemically smoothed Teflon, and the results of a trial showed that DLS had a longer patency period than standard polyethylene stents (with side holes) [19], although, in several cases of advanced bending of the bile duct, DLS of the proximal tip was found to be impacted on the bile duct wall, and this situation was suspected to be responsible for the reduced patency period. Nevertheless, the factor (Teflon or absence of side holes) that contributes most to favorable outcome remains unknown. Sung et al. showed in a randomized trial that polyethylene stents both with and without side holes perform equally well [20]. Recently, Van Berkel et al. compared polyethylene and Teflon Amsterdam-type stents in a randomized trial and could not find any difference in patency [21]. The combination of Teflon material and the absence of side holes results in superior patency rates, whereas omitting side holes in the design of a polyethylene stent or the use of Teflon material in a conventional design does not improve stent patency. Although the results of such clinical studies are contradictory, they emphasize that the smoothness of the stent surface is of great importance, either as a result of omitting side holes or by using material with a low friction coefficient. Thus, many trials have been undertaken in an effort to decrease the rate of clogging in plastic biliary stents. In these studies, the effectiveness of different stent materials and designs (with and without side holes, coated and uncoated) has been evaluated [17, 20–22]. A number of studies have shown that SEMSs have longer patency and lower occlusion rates than conventional PSs [1, 23–27]. CMSs have been developed recently to prevent tumor ingrowth and have shown a longer patency period than that of conventional UMSs [28–30]. Isayama et al. conducted a prospective randomized study of covered (handcrafted) versus uncovered diamond stents for the management of distal malignant biliary obstruction. The covered diamond handcraft stent prevented tumor ingrowth and was superior to the uncovered stent for the treatment of patients with distal malignant biliary obstruction [31, 32]. UMS has a self-expansive, metal wire mesh structure. Although cholecystitis and pancreatitis are rare, tumor ingrowth is a feared complication of both conditions, and while CMS might prevent tumor ingrowth, there is still a concern that the SEMSs covering the membrane might obstruct the pancreatic or cystic ductorifices, resulting in acute pancreatitis or cholecystitis. Additionally, various studies have shown the effectiveness of endoscopic removal of CMS using a snare [33]. Advanced bending of the bile duct and biliary obstruction near the papilla appears as inward migration. A recent study showed that uncovered Wallstents and covered Wallstents made no difference to stent patency. Because of consecutive inward migration using CMS in our department in 2001, we utilized UMS for subsequent cases of malignant biliary obstruction [34, 35]. In cases with short life expectancy (4–6 month or less), such as those with advanced pancreatic cancer, occlusion of PSs does not usually occur before death, and application of PSs is considered adequate from the viewpoint of cost-effectiveness [23, 25, 36–38].

The findings of this retrospective study revealed that because the conventional DLS and new DLS are made of the same materials and have the same shapes, only the presence or absence of side holes in the new DLS can be responsible for prolonging stent patency. Coene et al. also emphasized that when side holes are required in plastic stents to optimize proximal obstructions, their construction must be improved to ensure minimal inner wall irregularity. Therefore, we focused on inner wall smoothness and a longer patency period for the DLS [13].

The new DLS structure is the same as that of the conventional DLS, which has an inner layer made of PFA containing specially processed and chemically smoothed Teflon. The inner surface is smooth and does not undulate, which prevents the stent from becoming encrusted and clogged with biliary constituents or proteins [3, 4, 9].

In addition, the new DLS is modified with side holes. Although the previous side-hole stents had only one hole (3 mm) at both ends of the endoprosthesis, the new DLS has two holes (18 gauge) on the proximal side. The additional side hole may affect the patency period.

Moreover, this clinical study showed that the difference between new DLS and uncovered SEMSs had no effect on stent patency. New chemotherapeutic agents such as GEM are more effective for pancreatic cancer, and the survival time has improved [39]. In addition to its overall anticancer effects, stent patency should be prolonged by the suppression of tumor ingrowth and overgrowth. Stent occlusion may necessitate postponement of GEM administration and requires additional intervention or hospitalization which leads to deterioration of the patient's quality of life. Possible influences of GEM on the patency of stents are prolongation of patency by controlling the tumor mass and shortening of patency by clogging following biliary infection. It is expected that patients who undergo GEM will have a longer stent patency than those in non-GEM groups. The significance of stent patency maintenance is much greater than before as it can eliminate readmission due to stent occlusion and postponement of GEM. Further development of effective chemotherapy will further extend the significance of longer stent patency, and consequently, the selection of stents should be reassessed from this point of view.

Although various stents have been developed, simple stent placement and exchange, stent patency for more than six months, and low-priced stents are finally available. Therefore, when starting chemotherapy, we suggest the new DLS as the first-choice option.

This retrospective study showed the efficacy and safety of the new DLS with side holes, which is characterized by a high-cost-benefit ratio and may be considered the first choice for unresectable distal malignant biliary obstruction in cases with short life expectancy. However, further studies including cost-benefit assessment and a randomized, prospective comparison trial with side-hole DLS and CMS need to be conducted. To establish the clinical efficacy of the new DLS, we plan to conduct a prospective study of new the DLS versus the covered wall stent.

## Figures and Tables

**Table 1:** Patient characteristics by stent type and laboratory values on admission.

	New DLS	DLS	UMS
Number	23	24	17
Gender (m/f)	16/7	19/5	9/8
Age	68.1 ± 12.8	70.9 ± 11.2^∗1^	72.1 ± 11.2^∗2^
Diagnosis			
Pancreatic cancer			
III	2	4	2
IVa	6	6	7
IVb	11	11	4
Biliary duct cancer	0	0	2
Other	4	3	2
Unresectable due to:			
Advanced stage	21	20	13
Patient age	0	4	2
Other	2	0	2
Chemotherapy			
+	16	11	8
−	7	13	9
T-Bil (mg/dL)	6.8 ± 5.6	5.2 ± 4.6^∗1^	6.6 ± 7.4^∗2^
ALP (IU/L)	1027 ± 515	1296 ± 890^∗1^	1094 ± 910^∗2^

^∗1^New DLS+ versus DLS n.s. ^∗2^New DLS versus UMS n.s.

**Table 2:** Incidence of stent malfunction.

	DLS (24)	New DLS (23)	UMS (17)
Mean patency time	74 ± 66	160 ± 136^∗1^	196 ± 147^∗3^
Stent malfunction	16	15^∗2^	7^∗3^
(%)	(66.6)	(65.2)	(41.1)
Ingrowth	0	0	4
Overgrowth	2	1	0
Sludge	5	3	0
Food scraps	2	4	1
Impaction (bile duct)	3	3	0
Impaction (duodenal wall)	3	1	1
Other	1	3	1
Complication	3	2^∗2^	0^∗3^
(%)	(12.5)	(8.7)	(0)
Cholecystitis	2	0	0
Pancreatitis	1	0	0
Hemorrhage	0	0	0
Migration	0	1	0
Other	0	1	0

DLS versus new DLS: ^∗1^ *P* < 0.05. ^∗2^n.s. UMS versus new DLS: ^3^n.s.

**Table 3:** Risk factors for stent occlusion by univariate analysis.

	HR (95% CI)	*P*
Age (>65 versus 65 years)	0.951 (0.637–1.419)	0.507
Gender	0.910 (0.600–1.380)	0.427
Pancreatic cancer stage	1.348 (0.904–2.011)	0.100
IVb chemotherapy group	0.945 (0.651–1.370)	0.483
DLS	0.938 (0.647–1.359)	0.476
New DLS	0.877 (0.609–1.262)	0.341
UMS	1.286 (0.789–2.097)	0.205

CI: confidence interval; HR: hazards ratio.
